# An Embedded Real-Time Red Peach Detection System Based on an OV7670 Camera, ARM Cortex-M4 Processor and 3D Look-Up Tables

**DOI:** 10.3390/s121014129

**Published:** 2012-10-22

**Authors:** Mercè Teixidó, Davinia Font, Tomàs Pallejà, Marcel Tresanchez, Miquel Nogués, Jordi Palacín

**Affiliations:** Department of Computer Science and Industrial Engineering, University of Lleida, Jaume II, 69, 25001 Lleida, Spain; E-Mails: mteixido@diei.udl.cat (M.T.); dfont@diei.udl.cat (D.F.); tpalleja@diei.udl.cat (T.P.); mtresanchez@diei.udl.cat (M.T.); mnogues@diei.udl.cat (M.N.)

**Keywords:** real-time embedded system, autonomous system, fruit detection, LUT models

## Abstract

This work proposes the development of an embedded real-time fruit detection system for future automatic fruit harvesting. The proposed embedded system is based on an ARM Cortex-M4 (STM32F407VGT6) processor and an Omnivision OV7670 color camera. The future goal of this embedded vision system will be to control a robotized arm to automatically select and pick some fruit directly from the tree. The complete embedded system has been designed to be placed directly in the gripper tool of the future robotized harvesting arm. The embedded system will be able to perform real-time fruit detection and tracking by using a three-dimensional look-up-table (LUT) defined in the RGB color space and optimized for fruit picking. Additionally, two different methodologies for creating optimized 3D LUTs based on existing linear color models and fruit histograms were implemented in this work and compared for the case of red peaches. The resulting system is able to acquire general and zoomed orchard images and to update the relative tracking information of a red peach in the tree ten times per second.

## 1. Introduction

In recent years, machine vision has been used in many agricultural applications that require visual inspection and color grading systems. Probably one of the most promising pending future applications is the development of automatic and suitable harvesting machines for fresh fruit because the cost of the manual harvesting, that represents more than the 45% of the overall production cost in developed countries [1]. For example, in a typical agricultural area such as Lleida, in the autonomous region of Catalonia within Spain, and where our university is located, the production of Paraguayo peaches was 47,000 tones in 2011 and the forecast for 2012 is 66,300 tones, which represent a 41% annual increase [1]. In such an environment, the development of automatic harvesting machines specialized in fresh fruit is expected to have a large impact in the local and national economy by contributing to reducing the fruit production costs and also increasing the quality of the harvesting process. The development of suitable harvesting machines requires the combination of highly robotized picking machines with highly portable and compact autonomous vision systems to guide the automatic selection and picking of the optimal fruit [2,3].

The new contribution of this work is to propose the development of an autonomous real-time embedded vision system for fast fruit detection and tracking in acquired RGB color orchard images. The design and development of the embedded system is inspired in [4] and based on the preliminary work performed in [5]. In the future, this embedded system will be placed directly in a gripper tool of a robotized arm designed for automatic harvesting and the fully autonomous machine will be able to operate different arms simultaneously. Additionally, this work proposed the use of a three-dimensional look-up-table (LUT) defined in the RGB color space to perform fruit detection and tracking in the embedded vision system. Two methodologies were proposed and tested to create and optimize the LUT for the case of detecting and tracking red peaches: one based on 3D linear color models [6] and the other based on a 3D histogram [7]. At this moment, both methodologies proposed to create the LUTs used in the proposed embedded system require the manual selection of different image features, such as individual fruit and leaves in different orchard images, an aspect that must be automated in the future. However, once created, a 3D LUT defined in the RGB color space enables very fast fruit segmentation just by using the RGB pixel color intensities as table indices of the LUT because the value read from the table is directly the segmentation result.

## 2. Background

The background of this work refers mainly to the design of embedded vision systems and the application and benefits of the use of LUTs for image segmentation.

### 2.1. Embedded Vision Systems

Recent advances in computers and the improvement in micro-fabrication technology have fostered the design of tiny embedded systems with onboard electronics and sensors that also incorporates image-processing capabilities. For example, in [4], a versatile low cost embedded vision platform was presented to find blobs of a specific color in an image and also perform JPEG compression, frame differentiation, edge detection, image convolution, face detection, and color histogram computation. This embedded system was based on a low-cost CMOS color camera module, a frame buffer chip, and a low-cost microcontroller that processes all the images acquired and which was the inspiration for this work. Additionally, in [8], an intelligent embedded vision system to implement different image filters and perform image correlation and transformation at up to 667 frames per second was proposed. In [9], an embedded vision system was proposed specifically for mobile robot navigation and low power consumption. This embedded system accelerates the basic image processing algorithms required in a mobile robot application, such as low-level image processing, spatial filtering, feature extraction, and block matching operations. In [10], a FPGA was proposed to process the images from a CMOS camera to create an embedded and autonomous image processing system. In [11], an FPGA was also proposed for integrated navigation by combining GPS, gyroscopes, and vehicle odometry. The proposal in [12] was an embedded palmprint recognition system based on the dual-core OMAP 3530 platform to achieve real-time performances. In [13], an embedded vision system was proposed for intelligent driver nighttime assistance and surveillance. The system integrated different devices in order to analyze nighttime vehicle detection, collision warning determination and traffic recording.

### 2.2. Look-Up Tables for Fruit Segmentation

In a general application, fruit can be categorized by using different color features, for example by defining RGB thresholds [14–16], by analyzing multispectral images [17], by computing the color distance to reference colors [18], by measuring color characteristics [19], by applying fuzzy logic [20] or neural networks [21], or by using LUTs [22,23]. The general use of LUTs has the advantage of reduced run-time computations because of the transformation of the input data into an output value of a range of index values. This LUT-based transformation is very fast and the only drawback is the amount of memory required, which can be especially large in the case of multidimensional LUTs. In this particular work, fruit segmentation is performed by analyzing color features using a LUT in the proposed embedded system. Alternative shape based features, such as the circular Hough transform [24], or a combination between shape and color features [25] will be used in future works for fruit segmentation and detection.

In image-processing applications, LUTs are mostly used in the recognition of colored objects and are widely applied in embedded vision systems. For example, in [26], a three-dimensional RGB color LUT was proposed to segment similar colors in a robotic soccer scenario; a LUT with a memory space of 32 MB was required to classify any pixel image into four classes: field, yellow goal, blue goal and the ball, discarding the white pixels and classifying any other colors as obstacles. Finally, in [27], a system was proposed to sort olives automatically with a vision machine. In this case an operator creates a LUT for automatic olive segmentation by selecting representative samples of background, skin or blemish. The automatic system was able to process up to 396 olives per second, a rate that is impossible to achieve with a human operator.

## 3. Embedded Vision System

**Figure 1.**
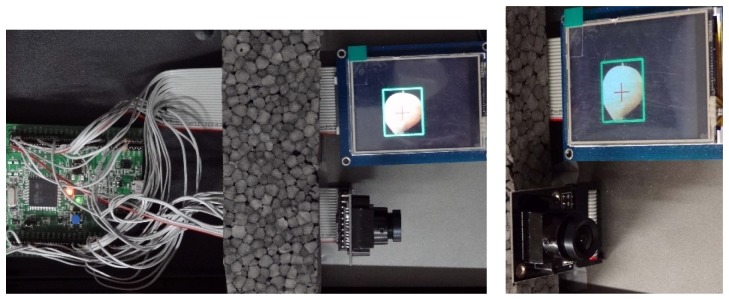
The camera module, the board processor and the auxiliary color LCD.

The embedded vision system used in this work was first proposed as a preliminary work in [5]. The proposed embedded system is based on an Omnivision (Santa Clara, CA, USA) OV7670 [28] camera and an STM32F407VGT6 [29] 32-bit microcontroller based on the ARM Cortex™-M4 processor developed by ARM [30] (Cambridge, UK) and manufactured by STMicroelectronics (Rennes, Switzerland). Figure 1 shows the complete embedded system containing the processor module, the camera, and an auxiliary color LCD that can be plugged in to obtain fast feedback from the embedded system. The communication between the embedded system and other external computers is performed by defining a standard client USB Communication Device Class (CDC). This is a generic client USB class that is identified by other computers as a new virtual serial port operated as a standard RS232 communications port.

### 3.1. Camera Module

The camera module, based on the Omnivision OV7670 color camera [28], is connected directly to a AL422B first in first out (FIFO) DRAM memory with 384 kB. Images are stored in the memory without any microcontroller supervision and can be later accessed sequentially. An external interruption is generated when a new image is available in the FIFO memory from the camera's VSYNC (vertical synchronism) signal. Then, the memory remains blocked until an external signal resets a flag to enable the acquisition of a new image. The image is accessed sequentially, byte by byte, starting in the first address of the FIFO and then, each subsequent reading increases an internal address counter. This counter can be reset by means of an external signal to allow multiple reading of the same image.

**Figure 2.**
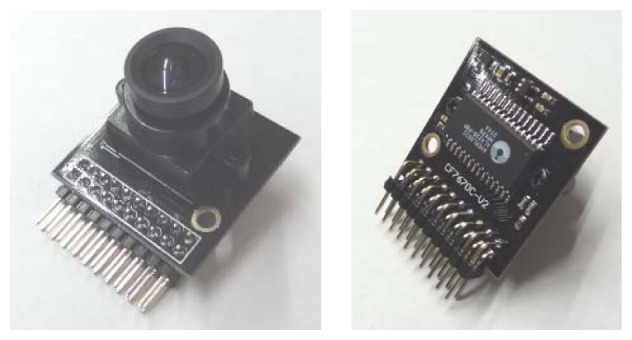
The camera module with the FIFO memory at the back.

The OV7670 camera is controlled through a serial camera control bus (SCCB) with full user control over image parameters such as color saturation, hue, gamma, sharpness, edge-enhancement, anti-blooming and also with automatic image quality functions. The camera can acquire images up to 640 × 480 active pixels (VGA size) in up to 30 frames per second (fps) in the standard YUV422, YCbCr422, RGB565, RGB555, GRB422, raw RGB, and raw Bayer RGB data formats. The camera can be configured to acquire images in VGA, CIF (352 × 288 pixels) and any resolution lower than CIF. It has an array size of 656 × 488 pixels with a pixel size of 3.6 μm × 3.6 μm. The camera requires voltages from 1.8 to 3.0 V and has a reduced power consumption of 60 mW. Finally, the camera has a sensitivity of 1.3 V/lux·sec, an automatic flicker detection system and a flash strobe output that can be used to control and synchronize external LEDs or other dedicated illumination systems, a feature that can be especially useful if the embedded system has to operate at night or in low light conditions. Figure 2 shows the camera and the FIFO module.

In this current application, the camera was finally configured to obtain images with a resolution of 320 × 240 pixels (QVGA size) with 16 bits/pixel in the RGB565 image format (5 bits for the Red color, 6 for the Green color, and 5 for the Blue color). This format only requires 150 kB of memory per image, allowing the storage of up to 2 images in the FIFO and also the storage of one complete image in the internal SRAM processor memory (with 196 kB). After segmentation, the resulting logical image only requires 9.4 kB of memory, or 75 kB if using a faster access byte codification.

### 3.2. Processor Board

**Figure 3.**
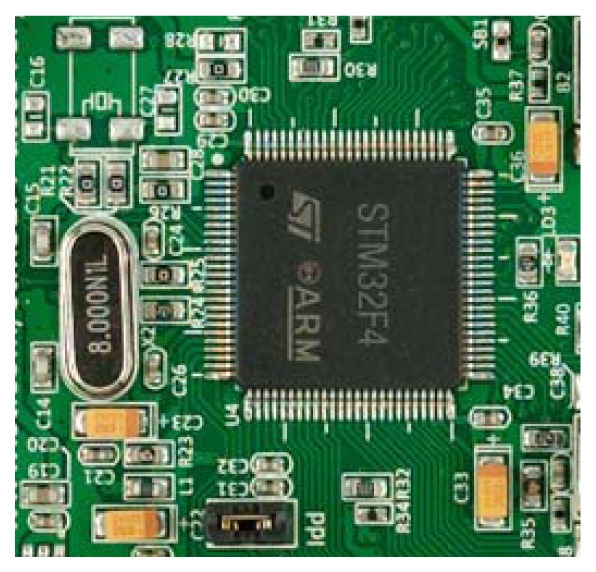
The processor used in the embedded system.

The processor board, based on the STM32F407VGT6 [29] (see Figure 3) processor can operate internally as a digital signal processor (DSP) and has a single-precision floating-point unit (FPU) allowing operations entirely done by hardware in a single cycle for most of the instructions. The microcontroller was configured to use an external crystal oscillator of only 8 MHz (see Figure 3) that is internally converted into an operative frequency of 168 MHz to operate at up to 210 Dhrystone MIPS.

According to the manufacturer's datasheet, the selected microcontroller dissipates only 139 mW when running at 100% CPU load, which makes it particularly interesting for use in embedded and portable devices. The microcontroller requires voltages from 1.8 to 3.6 V that are obtained from the 5 VDC of a USB client connection used to plug in the embedded system. The processor board has two USB 2.0 On The Go (OTG) serial buses that can be configured either as host or clients, an internal 10/100 Ethernet MAC with dedicated DMA, a configurable 8- to 14-bit parallel camera interface that can operate at up to 54 megabytes per second (MBps), several general-purpose DMAs, and up to 15 additional communications interfaces (I2C, UART, USART, SPI, CAN and SDIO). The processor board has 1 megabyte (MB) of flash memory, 192 + 4 KB of SRAM including 64-KB of Core Coupled Memory (CCM) data RAM, and a flexible static memory controller supporting Compact Flash, SRAM, PSRAM, NOR and NAND external memories. The USB client used to externally access the embedded system operates at 12 megabits per second (Mbps) (USB Full-Speed). The USB client, configured as CDC (virtual serial port), always operates at the same speed regardless of the serial speed used to open this virtual port. The communication is at the same speed even if the serial communication is configured at 9,600 or 115,200 bits per second (bps), although the real data throughput is far from the 12 Mbps due to the packet complexity of the USB communication.

## 4. Creation of a 3D Look-Up Table for Red Peach Detection

This section compares two methods that can be used to create a 3D LUT optimized for red peach detection and tracking. The first methodology is based on the previous definition of 3D linear color models [6] and the second on the prior definition of a 3D histogram [7]. In both cases, a manual selection of different image features, such as individual fruit and leaves, in different orchard images must be performed.

In general, a three dimensional LUT designed just to segment one object (the red peaches) in a conventional RGB color image with a color depth of 24 bits per pixel can be performed by defining a logical matrix with 256 × 256 × 256 × 1 bits (2 MB), but this LUT tends to be too large to be managed by an embedded system. Alternatively, the selected OV7670 camera operates directly in the RGB565 format with a color depth of 16 bits, enabling the definition of a reduced logical segmentation matrix with 32 × 64 × 32 × 1 bits (8 KB) that can be easily managed and included either in the internal Flash or SRAM memory of the onboard processor.

### 4.1. LUT Created from Linear Color Models Defined in the RGB Vector Color Space

The use of linear color models to detect red peaches in orchard images was proposed in [6]. The creation of linear color models in the RGB vector color space requires expert manual operation and the selection of the area of the different objects of interest in the images: fruit, leaves, branches, *etc*. The general use of linear color models has the drawbacks that all objects of interest must be modeled and that most objects may require the definition of more than one linear color model to cover all variance in object illumination, such as brightly illuminated peach, dark peach, yellowish peach and brownish peach (see [6] for more details). However, the use of linear color models has the advantage that the color evolution of one object within a region is modeled with a regression line that can predict (extrapolate) unknown object color relationships, reducing the number of training images required to create and tune these linear color models.

**Figure 4.**
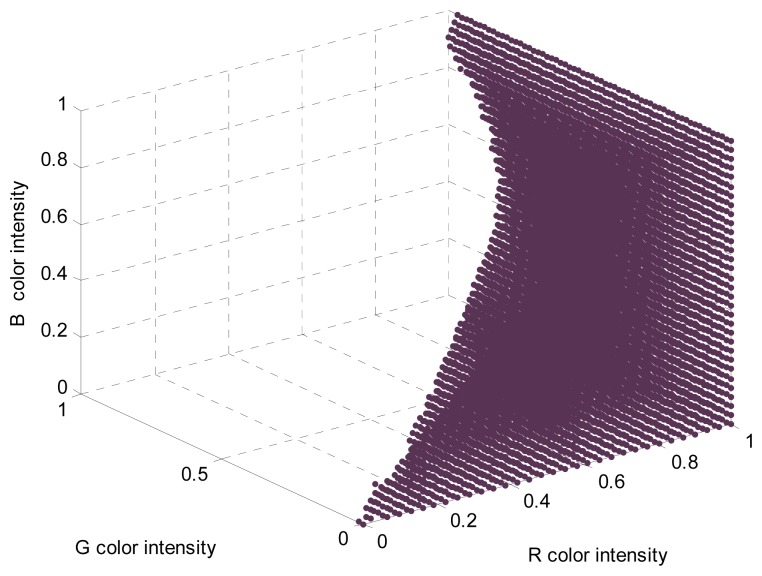
Red peach segmentation LUT obtained from linear color models.

The different linear color models of the elements of the orchard can be converted into a segmentation LUT also defined in the RGB vector color space just by classifying any [R,G,B] pixel color intensity as a member of the class “red peach” (labeled as “1” in the LUT) or not (labeled as “0” in the LUT); the pixel color intensities will also be the indices of the LUT. This pixel classification can be very time consuming depending on the number of linear color models available but only has to be performed once; the resulting 3D LUT will then summarize all object color knowledge described previously with the linear color models of the orchard. Figure 4 shows the red peach segmentation LUT obtained from the linear color models created to detect red Paraguayo peaches in orchard images (repeating the process described in [6]). This LUT was created with 32 levels in the R index color vector, 62 in the G index color vector, and 32 in the B index color vector to be directly implemented in the proposed embedded system to segment the RGB565 images acquired by the onboard camera.

### 4.2. LUT Created from the Histogram Defined in the RGB Vector Color Space

The alternative method proposed to create a 3D LUT to segment red peaches in the RGB vector color space is based on the creation of a three-dimensional histogram with the frequency of the different pixel [R,G,B] color intensities of red peaches (see [7] for a useful introduction to three-dimensional histogram color based object detection). The creation of the three-dimensional histogram in the RGB vector color space also requires a manual operation to select the area of several red peaches appearing in different images and illumination conditions. The general use of a three-dimensional histogram to classify individual pixels has the drawback that a large image database is required to cover all object pixel color variance, although the management of these images is very simple and limited to the selection of the object of interest in different images.

The three-dimensional histogram can be converted into a segmentation LUT also defined in the RGB vector color space simply by segmenting the histogram. This process is faster than the previous method because there is no need to classify all possible color intensity combinations. The segmentation process is as follows: any [R,G,B] pixel color intensity combination with a probability higher than a threshold (zero in this case) is labeled as “1” in the LUT and labeled as “0”otherwise; the pixel color intensities are also the indices of the LUT.

**Figure 5.**
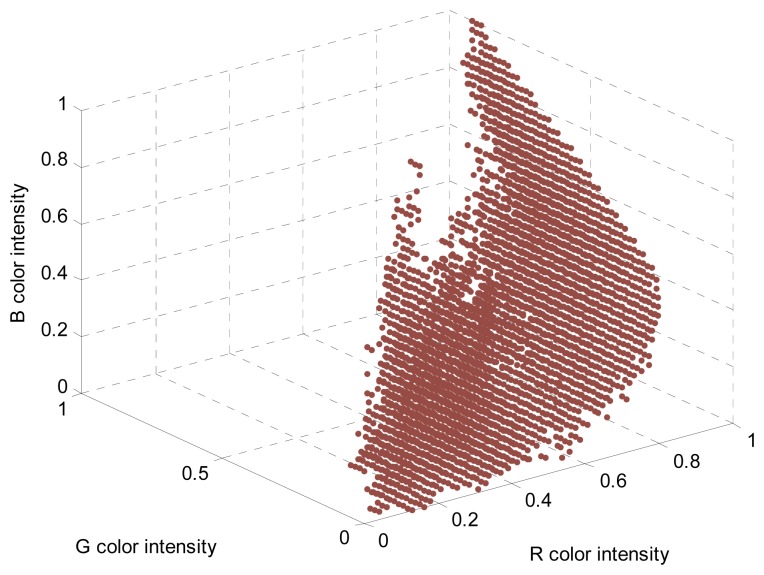
Red peach segmentation LUT obtained from a three-dimensional histogram.

The resulting 3D LUT will summarize all object color knowledge described previously with the three-dimensional histogram. Figure 5 shows the red peach segmentation LUT obtained from the three-dimensional histogram created by selecting the area corresponding to red peaches in different images of an orchard. This LUT was created with 32 levels in the R index color vector, 62 in the G index color vector, and 32 in the B index color vector to directly segment the RGB565 images acquired by the onboard camera of the embedded system.

## 5. Experimental Results

This section is focused on evaluating the fruit detection performances of the proposed embedded system. The red peach detection will be performed with the two LUTs proposed in the previous section and their detection performance is compared. The detection experiments were carried out with the embedded system powered by a conventional USB connection (5 VDC). The intensity required by the embedded system in normal operation with the processor operating at full speed and the LCD active was only 210 mA.

The embedded system's camera is configured to operate at 30 fps so it generates a new image every 33.3 ms that can be stored directly in the FIFO buffer if this is enabled to receive and store the image. Any image stored in the FIFO must be read sequentially, starting in the first position of the FIFO (first pixel of the image) and ending in a position that will correspond to the last byte of the image. By resetting the internal “reading counter” flag of the FIFO, the stored image can be reread (sequentially) several times. The occupancy of the FIFO will change depending on the image resolution, image format, and digital zoom applied (the limit is 384 Kbytes).

**Figure 6.**
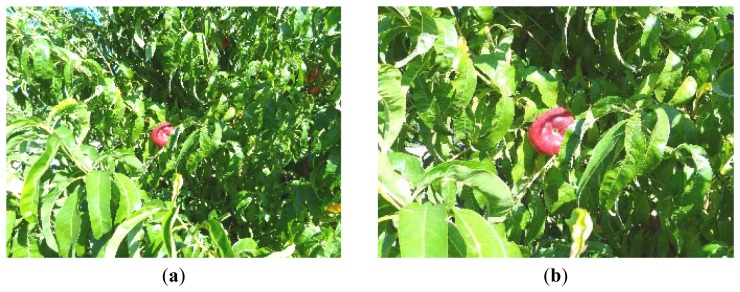
Orchard image (320 × 240 pixels, RGB565) obtained in normal (**a**) and zoom mode (**b**).

To increase the flexibility of the embedded system, the camera format was configured to operate at a resolution of 320 × 240 in the RGB565 format pixels and in two complementary modes: normal mode and zoom mode, in both cases the size of the image acquired will be 150 Kbytes. The camera has an internal matrix of 640 × 480 active pixels and uses a Bayer filter to generate the RGB color codification of the images. In the normal mode (Figure 6(a)) the camera is requested to submit 320 × 240-pixel default images and it offers this image just by sampling (by a factor of 2) the rows and colors of a base 640 × 480 RGB image. This mode offers an image that covers the full angle of view of the lens available on the camera. In the zoom mode (Figure 6(b)), the starting and ending rows and columns of the resulting image can be configured to generate an effective sequential window of 320 × 240 pixels with a narrow angle of view that can be displaced along the original 640 × 480 image matrix of the image. This zoom mode can be selected after processing and selecting one fruit in the normal image to increase the resolution of the image and the accuracy of the detection (see Figure 6).

### 5.1. Evaluation of the Time Performances

Table 1 summarizes the time spent by the proposed embedded system to develop different segmentation alternatives applied to one orchard image (resolution 320 × 240, format RGB565, normal mode without zoom) in order to detect the red peaches in the image. The first algorithm only refers to the time required to access the image in the FIFO memory and copy it to the processor memory without any change. This time is very long and limits the real frame rate to 10 fps (the camera generates images at intervals of 33.3 ms so only one of each three frames can really be processed). The next algorithms reveal the time spent on a simple one layer threshold segmentation, in a threshold segmentation applied to the three color layers and in interval segmentation applied to the three color layers, in all these cases the real frame rate that can be achieved is again 10 fps. The algorithm labeled as “64 kB LUT segmentation” refers to the use of the 16 bits of each RGB565 pixel color value as the index of a byte position in the LUT (32 × 64 × 32 × 1 byte). This byte-based LUT requires 64 kB of memory but can be addressed faster than a bite-based LUT. The algorithm labeled as “8 kB LUT segmentation” refers to the optimal LUT implementation by using 13 bits of each RGB565 pixel color value as the index of a byte position in the table (4 × 63 × 32 × 1 byte) and the use of the remaining 3 bits as the index to the bit value of the LUT. This bit-based LUT only requires 8 kB of memory. The LUT defined in both cases is exactly the same, as is the application frame rate, so the selection will depend mainly on memory and speed limitations. The next row summarizes the time spent on the LUT based segmentation and in the later computation of the centroid of the red peaches segmented (labeled with a red cross in the images in Figure 1) that can operate at 10 fps, a high enough rate to guide future evolutions of a robotized arm in order to pick a selected fruit.

Finally, the last row of Table 1 shows the time spent by the embedded system when trying to classify each pixel by using the linear color models empirically proposed in [6]. In this case, the high number of linear color models defined to properly classify and segment the red peaches and the complexity of the mathematical operations required, most of them in floating point arithmetic, preclude the practical application of this procedure as the segmentation results are exactly the same as when using a segmentation LUT created previously from these linear color models.

### 5.2. Evaluation of Fruit Detection Performances

This section is focused on evaluating the fruit detection performances of the embedded system when using a red-peach segmentation LUT created either with a three-dimensional linear color model analysis of the images of the orchard or with a three-dimensional histogram. The methodology proposed to evaluate these performances is as follows: (1) the image of the orchard is acquired by the camera of the embedded system; (2) the image is stored for offline analysis; (3) the image is segmented by using the logical LUT created in the previous section from existing linear color models and stored for offline analysis; (4) the original image is segmented again by using the logical LUT created in the previous section from the three-dimensional histogram. Then, the offline analysis performed to compare both segmentation results is as follows: (1) the red peaches in the original orchard image are manually labeled; (2) both the automatically-segmented images are compared with the manual segmentation to evaluate the differences. Tables 2 to 5 summarize the results obtained from the analysis of 100 different orchard images where red peaches have different illumination and occlusion conditions.

Table 2 shows the difference between the area of red peaches manually labeled and the segmentation obtained with both LUTs in the case of no occlusion in the peaches and bright natural illumination and low illumination. The table shows the average error expressed in pixels relative to the manual operation. The *LCM-LUT* label symbolizes the use of a segmentation LUT obtained from the application of linear color models and *Histogram-LUT*, the use of a segmentation LUT obtained from a three-dimensional histogram. In both cases, the average relative error found is very similar but slightly worse in the *LCM-LUT* case.

Table 3 shows the difference between the area of red peaches obtained with both LUTs in the case of bright illumination and peaches with different occlusions. Again, the results in both cases are very similar and both LUTs have similar problems to properly segment the skin of the peaches affected by the illumination changes caused by the occlusion. However, in this case, the results of the *LCM-LUT* are slightly better.

Table 4 shows the average noisy pixels that appear in the segmented image relative to a manually labeled image for different illumination conditions. These pixels are wrongly segmented as part of the red skin of the peaches and can affect further detection or localization algorithms. In this case, the results with the *LCM-LUT* are slightly better probably because the linear color models can predict pixel color relationships in areas without previous color information, whereas the histogram-based LUT is unable to perform this prediction in areas with no previous color information.

Finally, Table 5 shows the average noisy pixels that appear in the segmented image relative to a manually-labeled image for different occlusion conditions. In this case the results from the *LCM-LUT* are again slightly better probably because the use of three-dimensional linear color models is able to predict the color relationships and illumination changes that appear on the skin of red peaches affected by different degrees of occlusion.

## 6. Conclusions

This work proposes the development of an embedded real-time fruit detection system ready to be used in future robotized machines optimized for fruit harvesting. The embedded system is built around a state-of-the-art processor and a state-of-the-art miniaturized color camera. The selected processor requires very low power but operates at very high performance and is based on a constantly evolving ARM core proposal initially designed for the smartphone market. The selected color camera also requires very little power and generates high quality images under bright natural illumination. The camera has an internal image matrix of 640 × 480 pixels and can be configured to generate the 320 × 240 pixel images required by the system in a normal and zoom mode. Additionally, the use of specific image formats, such as the RGB565, limits memory occupancy to 150 KB and also reduces the memory occupancy of the segmentation LUTs used to detect red peaches in the images.

The validation experiments performed with the proposed embedded system have shown that the tracking of a red peach can be updated 10 times per second and that the use of different LUTs optimized for the RGB565 image format generates very few noisy pixels in the segmented image (less than 11%). In both cases, the area of a red peach was estimated with an average error of less than 10% when the piece of fruit is not affected by occlusion. This update time is very important because the embedded system is designed to be included in the gripper tool of a robotic arm to control the approximation of the gripper to the fruit while the harvesting machine is in movement. With this proposal, the gripper tool will be able to update its relative position with the target fruit 10 times per second. Finally, the experimental results obtained showed that a LUT created from previously defined linear color models has slightly better segmentation results as it can better estimate the skin color relationships originated in the red peach by illumination changes.

Future work will center on evaluating high-resolution megapixel miniature color cameras to explore the definition of different image zooming operations while maintaining the QVGA size of the images acquired and the operational speed of the embedded system. Future work will also be focused on miniaturizing the embedded system in order to incorporate the system directly onto the gripping tool of a robotized arm designed for fruit harvesting.

## Figures and Tables

**Table 1.** Segmentation algorithm, time required to perform the operations and the real frame rate achieved.

***Algorithm***	***Time (ms)***	***fps***
Image copy from the FIFO to processor memory	40.30	10
Image reading from the FIFO + Threshold segmentation of 1 color layer [example: R > 16] + Result stored into processor memory	44.28	10
Image reading from the FIFO + RGB threshold segmentation [example: R > 16 & G < 55 & B < 12] + Result stored into processor memory	46.61	10
Image reading from the FIFO + RGB interval segmentation [ex.: 32 > R > 12 & 55 > G > 10 & 16 > B > 12] + Result stored into processor memory	52.23	10
Image reading from the FIFO + 64 KB LUT segmentation using the RGB565 16 bits value as an index [ex.: s = LUT(RGB565)] + Result stored into processor memory	44.41	10
Image reading from the FIFO + 8 KB LUT segmentation using 13 bits as a pointer and 3 bits as byte offset [ex.: s = LUT(RGB565(1:3), RGB565(4:16),)] + Result stored into processor memory	51.73	10
Image reading from the FIFO + 8 KB LUT segmentation using 13 bits as a pointer and 3 bits as byte offset [ex.: s = LUT(RGB565(1:3), RGB565(4:16),)] + Result stored into processor memory + Red Peach centroid computation	63.64	10
Image reading from the FIFO + Linear Color Model classification and segmentation (11 classes) + Result stored into processor memory	2,228.47	0.44

**Table 2.** Average relative error in the area estimate of red peaches for different illuminations.

***Light Conditions (No Occlusion)***	***LCM-LUT***	***Histogram-LUT***

Bright Illumination	9.86%	6.42%
Low Illumination	7.89%	7.59%

**Table 3.** Average relative error in the area estimate of red peaches for different occlusions.

***Occlusion Conditions***	***LCM-LUT***	***Histogram-LUT***

Occlusion ratio lower than 33%	7.77%	7.80%
Occlusion ratio from 33% to 66%	12.43%	13.64%
Occlusion ratio from 66% to 99%	21.36%	23.20%

**Table 4.** Average noisy pixels in the segmented image for different illuminations.

***Light Conditions (No Occlusion)***	***LCM-LUT***	***Histogram-LUT***

Bright Illumination	3.40%	5.71%
Low Illumination	4.76%	7.81%

**Table 5.** Average noisy pixels in the segmented image for different occlusions.

***Occlusion Conditions***	***LCM-LUT***	***LUT Histogram***

Occlusion ratio lower than 33%	3.37%	5.99%
Occlusion ratio from 33% to 66%	5.51%	8.97%
Occlusion ratio from 66% to 99%	7.82%	10.87%
